# Structural similarity-based predictions of protein interactions between HIV-1 and Homo sapiens

**DOI:** 10.1186/1743-422X-7-82

**Published:** 2010-04-28

**Authors:** Janet M Doolittle, Shawn M Gomez

**Affiliations:** 1 Curriculum in Bioinformatics and Computational Biology, University of North Carolina at Chapel Hill, Chapel Hill, North Carolina, USA; 2 Department of Computer Science, University of North Carolina at Chapel Hill, Chapel Hill, North Carolina, USA; 3 Department of Pharmacology, University of North Carolina School of Medicine, Chapel Hill, North Carolina, USA; 4 Joint Department of Biomedical Engineering, University of North Carolina School of Medicine, Chapel Hill, North Carolina, USA

## Abstract

**Background:**

In the course of infection, viruses such as HIV-1 must enter a cell, travel to sites where they can hijack host machinery to transcribe their genes and translate their proteins, assemble, and then leave the cell again, all while evading the host immune system. Thus, successful infection depends on the pathogen's ability to manipulate the biological pathways and processes of the organism it infects. Interactions between HIV-encoded and human proteins provide one means by which HIV-1 can connect into cellular pathways to carry out these survival processes.

**Results:**

We developed and applied a computational approach to predict interactions between HIV and human proteins based on structural similarity of 9 HIV-1 proteins to human proteins having known interactions. Using functional data from RNAi studies as a filter, we generated over 2000 interaction predictions between HIV proteins and 406 unique human proteins. Additional filtering based on Gene Ontology cellular component annotation reduced the number of predictions to 502 interactions involving 137 human proteins. We find numerous known interactions as well as novel interactions showing significant functional relevance based on supporting Gene Ontology and literature evidence.

**Conclusions:**

Understanding the interplay between HIV-1 and its human host will help in understanding the viral lifecycle and the ways in which this virus is able to manipulate its host. The results shown here provide a potential set of interactions that are amenable to further experimental manipulation as well as potential targets for therapeutic intervention.

## Background

Pathogen invasion and survival requires that the pathogen interact with and manipulate its host. Human immunodefficiency virus type 1 (HIV-1) encodes only 15 proteins and must therefore rely on the host cell's machinery to accomplish vital tasks such as the transport of viral components through the cell and the transcription of viral genes [1,2]. HIV-1 infects human cells by binding to CD4 and a coreceptor, fusing with the cell membrane and uncoating the virion core in the cytoplasm [2]. The genomic RNA is then reverse transcribed and the DNA enters the nucleus as part of a viral pre-integration complex (PIC) containing both viral and host proteins. Afterwards, the viral DNA is inserted into the genome by viral integrase (IN) [1]. The integrated provirus is transcribed by host RNA polymerase II from a promoter located in the provirus long terminal repeat (LTR), and the RNA is exported to the cytoplasm [1,2]. Host machinery translates HIV-1 mRNA, and several of the resulting proteins are transported to the cell membrane to be packaged into the virion along with the genomic RNA and multiple host proteins. The virus then buds from the cell and undergoes a maturation process, which enables it to infect other cells [2]. Throughout this process, host proteins play an indispensable role.

To understand the interface through which the pathogen connects with and manipulates its host requires knowledge of the molecular points of interaction between them. Specifically, knowledge of the protein interactions between pathogen and host is of particular value. While the prediction of protein interactions within species such as *S. cerevisiae *and *H. sapiens *has been pursued for some time, it is only recently that host-pathogen interactions have come under greater scrutiny. Indeed, computational approaches are of significant value in the host-pathogen context as large-scale experimental characterization of these interactions is non-trivial [3-6].

As a result of the need for computational approaches, several recent methods have been developed and applied to host-pathogen interactions, suggesting additional potential interactions in different host-pathogen systems. For instance, Dyer et al. predicted interactions between *P. falciparum *and human using statistics about domains involved in within-species interactions [7]. Also focusing on malaria, Lee and colleagues generated predictions based on interactions between orthologous proteins from eukaryotes [8]. In the context of HIV-human interactions, at least two computational methods have been applied. In the first study, Tastan et al. used a computational approach based on the random forest method to predict protein interactions using features taken from human proteins and the human interactome [9]. In the second study, Evans et al. predicted possible interactions using short sequence motifs conserved in both HIV-1 and human proteins [10].

While of value, most approaches have not utilized the significant amount of protein structure information that is increasingly available. Specifically, rapid progress in structure determination technologies has led to the establishment and deposition of massive numbers of protein structures into the Protein Data Bank, with over 60,000 protein structures currently deposited [11]. In combination with documented protein-protein interactions, the use of protein structure information provides another means for the prediction of possible protein interactions [12-14]. The central premise in such approaches is that, given a set of proteins with defined structures and associated interactions, proteins with similar structures or substructures will tend to share interaction partners. In the context of host-pathogen interactions, Davis et al., used homology modeling to ascertain potential protein interactions for pathogens responsible for several tropical diseases [15]. Unfortunately, despite their potential value, such computational structure approaches have not been widely applied to the problem of predicting host-pathogen interactions.

**Figure 1:**
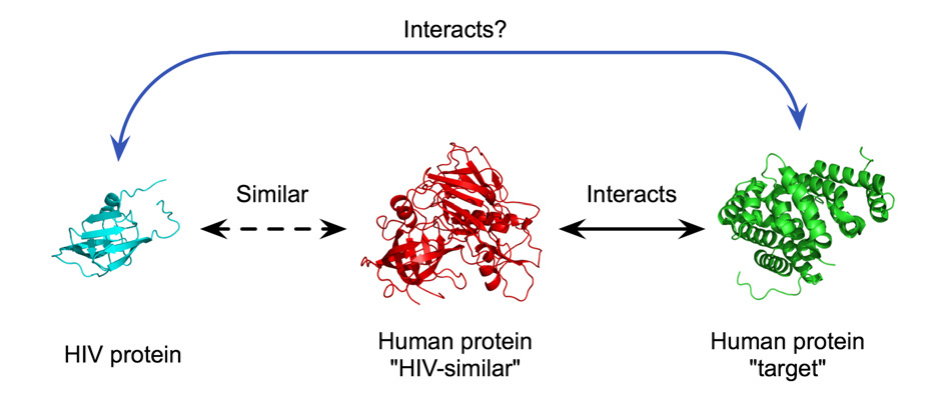
**Diagram of approach**. HIV-1 proteins showing structural similarity to one or more human proteins are first identified. Interactions for these "HIV-similar" proteins with other human proteins are then identified. Following appropriate filtering, this methodology predicts the existence of a physical interaction between the HIV protein and the human "target" protein(s).

Here, we develop a map of interactions between HIV-1 and human proteins based on protein structural similarity. In this approach, we first retrieve structural similarity between host and pathogen proteins identified by an established method which compares known crystal structures. Human proteins identified as having a region of high structural similarity to an HIV protein are referred to as "HIV-similar." Next, we identify known interactions for these HIV-similar proteins, with the one or more human proteins that they interact with referred to as "targets." We then assume that HIV proteins have the same interactions as their human, HIV-similar counterparts, allowing HIV to plug into the host cell protein network at these points (Figure 1). Using data from recent RNAi screens and cellular co-localization information, we refine this interaction map so as to enrich for those interactions having the greatest potential to be correct based on the available information. Evaluation of these predictions shows a statistically significant enrichment of known interactions as well as numerous novel interactions with potential functional relevance. These predictions provide an additional tool for further investigations into the lifecycle of HIV-1 and identification of potential clinical targets.

## Results and Discussion

### Identification of HIV-similar human proteins

**Figure 2:**
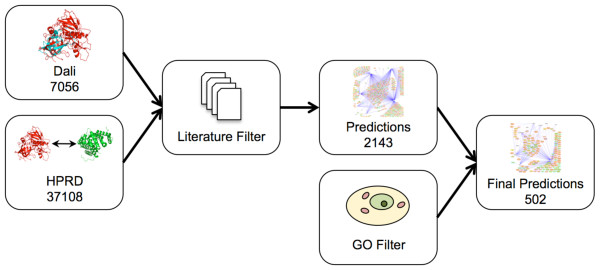
**Structural prediction workflow**. Structural similarities from Dali and known interactions between human proteins from HPRD are used to predict interactions between HIV-1 and human proteins. These predictions are filtered based on functional information from previous studies to make a first set of predictions. This set is further filtered using GO cellular component terms to yield a final prediction set including fewer predictions with higher confidence. Numbers represent the number of interactions, or structural similarities in the case of Dali, at each stage of the process.

To construct a map of interactions between HIV-1 and human proteins, we established a multi-step protocol that begins with the identification of human proteins having significant structural similarity to HIV-1 proteins (Figure 2). We used the Dali Database [16,17], which contains 3D structure comparisons for all protein structures in the Protein Data Bank (PDB); all publicly available crystal structures for HIV-1 and *H. Sapiens *are contained within PDB. While the crystal structure for many human proteins is unknown, most HIV-1 proteins have been at least partially resolved. Specifically, crystal structures exist for PR, RT, IN, CA, MA, NC, Gag p2, gp120, gp41, Nef, Tat, Vpr, and Vpu (Table 1). The three enzymes encoded by HIV-1, protease (PR), reverse transcriptase (RT), and integrase (IN) are the best characterized structurally, having at least 25 structures each in the PDB, with PR having over 300. CA, gp41, and gp120 are also fairly well studied. We note, however, that many of these structures represent only part of the full-length protein. HIV-1 proteins having regions of high similarity to at least one human protein include: gp41, gp120, CA, MA, Gag p2, PR, IN, RT, and Vpr (Additional File 1). Therefore, predictions were made for nearly every HIV-1 protein that has a published structure.

**Table 1:** HIV-1 protein structures

Representation of HIV-1 proteins		
**HIV-1 protein**	**PDB chains in Dali**	**PDB structures in Dali**

capsid	52	25

gp120	24	20

gp41	24	17

integrase	51	26

matrix	17	12

nef	5	3

nucleocapsid	3	3

p2	1	1

protease	604	304

reverse transcriptase	176	85

tat	3	3

vpr	1	1

vpu	1	1

The number of structures representing each HIV-1 protein in Dali.

**Figure 3:**
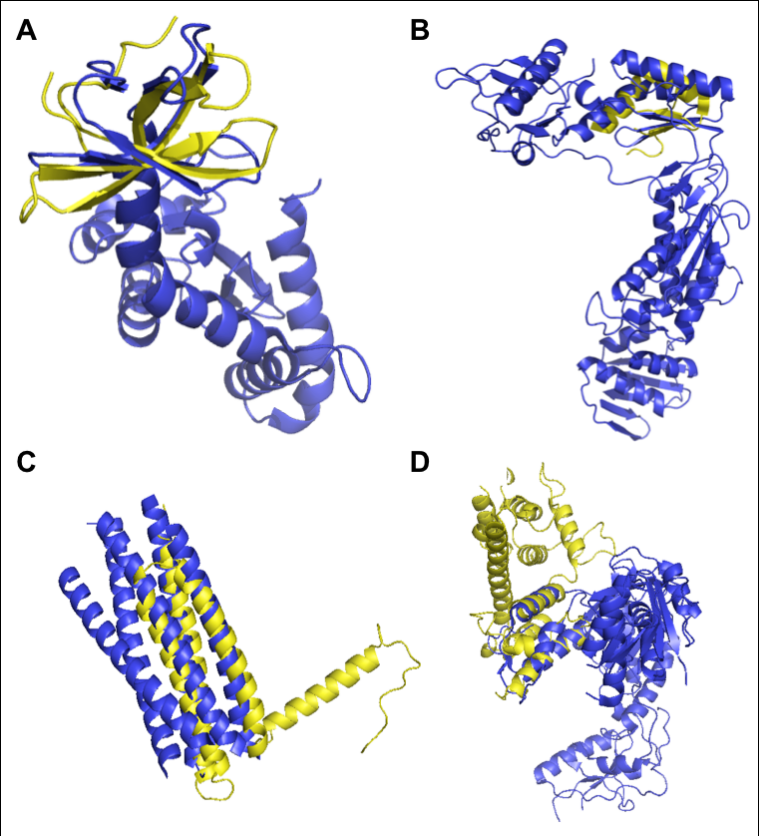
**Selected Structural Similarities**. Structures of HIV-1 and human proteins aligned using Dali. *(A) *IN (1ex4A) aligned with SMN2 (1g5vA) [51,52]. *(B) *NXF1 (1ft8E) aligned with RT (1tl3A) [53,54]. *(C) *gp41 (2cmrA) aligned with PTK2 (1k04A). [55,56]. *(D) *RT (1lwcA) aligned with PLEC1 (1mb8A) [57,58]. HIV-1 proteins are in blue, human proteins are in yellow.

Selected examples of structural similarities between the HIV-1 proteins IN, RT, and gp41 and human proteins determined by Dali are shown in Figure 3. The structural similarities frequently involve only part of each protein. However, since in most cases the precise location of protein interaction sites is not known, we used the entire structure in our investigation.

### Protein interaction prediction

Upon obtaining the knowledge of which specific HIV-1 and human proteins have high structural similarity, we extract all known interactions for human proteins from the Human Protein Reference Database, which contains over 37,000 documented protein interactions [18]. Again, the central premise is that given a network of protein interactions, proteins with similar structures or substructures will tend to have similar interaction partners. Thus, our hypothesis is that HIV-1 proteins having similar structure to one or more human proteins are also likely to participate in the same set of protein interactions (Figure 1). Under these assumptions, we directly mapped HIV-1 proteins to their high-similarity matches within this network. 

To reduce the number of predictions and provide an additional line of functional evidence for interactions and their possible biological relevance, we filtered these results using two types of datasets on host proteins involved in HIV-1 infection; collectively referred to as "Literature Filters" hereon. The first type represents host proteins that have been shown to impair HIV-1 infection or replication when knocked down by siRNA or shRNA. Three genome-scale siRNA screens have been conducted in HeLa or 293T cells [19-21]. A fourth study with a similar goal was conducted using shRNA in Jurkat T-cells, a more realistic model of HIV-1 infection [22]. Each of the four screens found over 250 host proteins involved in HIV-1 infection. Remarkably, very little overlap exists between these studies, perhaps due to differences in methods, including the cell lines and stages of the HIV-1 life cycle investigated.

The second type of data used to filter predictions is literature data identifying human proteins present in the HIV-1 virion. During budding, host proteins from both the cell surface and the cytoplasm, including some involved in the cytoskeleton, signal transduction, metabolism, and chaperones, may be incorporated into the virion [23]. While some of these proteins may be taken up by the budding virus simply by chance, others are known to be specifically incorporated into the virion and may play key roles in viral life cycle or pathogenesis. For example, TSG101 may be incorporated due to its interaction with Gag, and facilitates budding [23,24].

We considered only predicted interactions where the target protein was observed in at least one of the previously described Literature Filters. The resulting predicted HIV-human interaction network consists of 2143 interactions, considering all unique combinations of Uniprot accessions for an HIV-1 protein and a predicted human interactor (Figure 2). Of the predictions that were made, 62 were verified as true interactions based on data from two databases of known host-pathogen interactions, HHPID and PIG (Additional Files 2 and 3). There were 347 human proteins predicted to have structural similarities with an HIV-1 protein and the predictions implicate a total of 406 unique human proteins as potentially interacting with HIV-1 (Table 2).

**Table 2:** Summary of Predicted Interactions

Prediction Results Summary		
	**Before CC filter**	**After CC filter**

Structure Nodes	11	10

HIV-1 Uniprot	49	33

Similar Human Proteins	347	189

Predicted Human Binding Partners	406	137

True Positives	62	31

Total Predictions	2143	502

Percent True Positive	2.89%	6.18%

The number of proteins found as well as interaction predictions made by the method are shown. HIV-1 Structure Nodes refers to the number of HIV-1 proteins represented in Dali, while HIV-1 Uniprot refers to the number of HIV-1 Uniprot accessions present in the predictions. Human proteins and predicted interactions are counted by unique Uniprot accessions.

We visually examined some of the structural similarities that led to predictions that were already known. SMN2 is structurally similar to integrase (IN) (Figure 3A, Additional File 1) and both SMN2 and IN are known to interact with SIP1 (Gemin2) [18,25]. SIP1, part of the large SMN complex involved in the assembly of snRNPs, may also be part of the pre-integration complex during HIV-1 infection and may aid viral reverse transcription [26]. There are also several predicted interactions between IN and host proteins that interact with SMN2 that have not yet been tested (Additional File 1). The structural similarities shown in Figure 3B-D also led to predictions of known interactions, even though only part of the proteins are structurally similar.

### Protein co-localization

**Figure 4:**
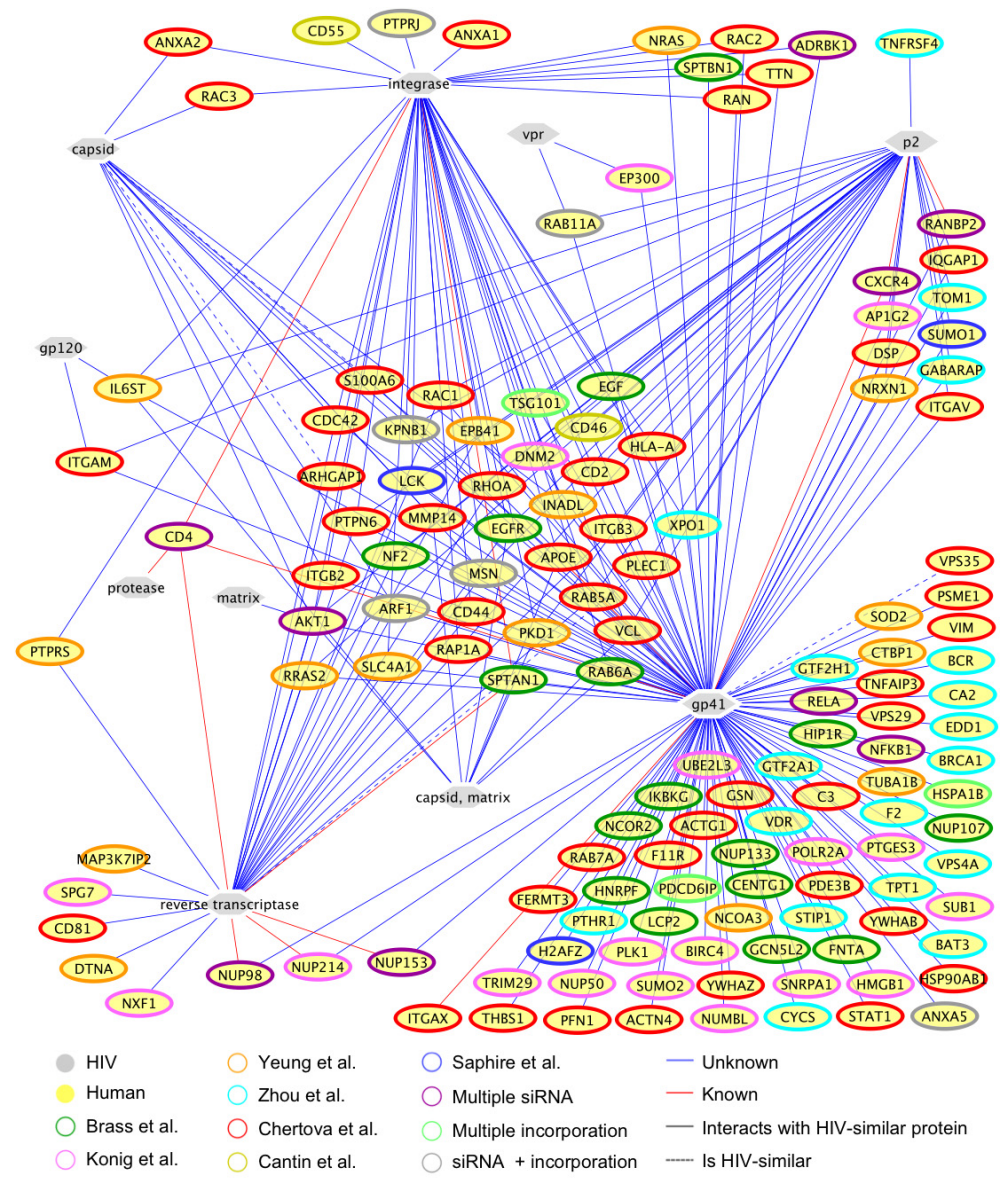
**Predicted interaction network after cellular component filtering**. In addition to the prediction of a physical interaction, the human proteins included in this prediction set are known to have a role in HIV-1 infection or replication as supported by 1) evidence of incorporation into the HIV-1 virion or 2) their reduced expression is known to prevent HIV-1 infection (node line color corresponds to source). Predictions were filtered to contain only those pairs of proteins that share at least one Gene Ontology cellular component term. Red lines represent predicted interactions that are already known to occur.

To further narrow the list of likely interactions, we refined these results by requiring both the HIV-1 protein and the target human protein to be present in the same location within the cell, based on GO cellular component (CC) annotation. The refined set of predictions is shown in Figure 4. Including this filtering step reduced the number of interaction predictions to 502, involving 189 HIV-similar proteins having 137 known different binding partners. There are 31 predictions corresponding to already known HIV-human interactions (Table 2, Additional File 4). Using the criterion that interacting proteins must have some evidence of co-localization not only reduced the size of the predicted interactome, but also increased the percentage of true positive predictions from ~3% true positives before filtering to over 6% after filtering (Table 2).

Taking localization into account, gp41 has many more predicted interactors than any other HIV-1 protein. This is most likely due to the relatively large number of GO cellular component terms that were annotated to gp41 and also relevant to the host cell. Since gp41 is known to be found in more parts of the cell than other HIV-1 proteins, a larger number of human proteins were able to meet the co-localization criterion.

The interaction predictions made by this method are specific for structures, and we note that different structures for a single protein may lead to different predictions about its interactions. Therefore, some information is lost if predictions are described at a gene level. Nevertheless, it may be of interest to consider interactions on a gene basis (See Additional File 5 for the mapping of HIV-1 IDs). When counted according to the HIV-1 protein node names and human target Entrez Gene IDs, we made 883 interaction predictions, 56 of which were true positives according to HHPID and PIG. Following CC filtering, we had 22 true positive predictions among 265 total predictions (~10% of known true positives). While these results tend to suggest higher rates of predictive accuracy when using our method, we report our more conservative Uniprot-based accuracy values as our best estimates.

### Properties of human proteins predicted to interact with HIV-1

**Figure 5:**
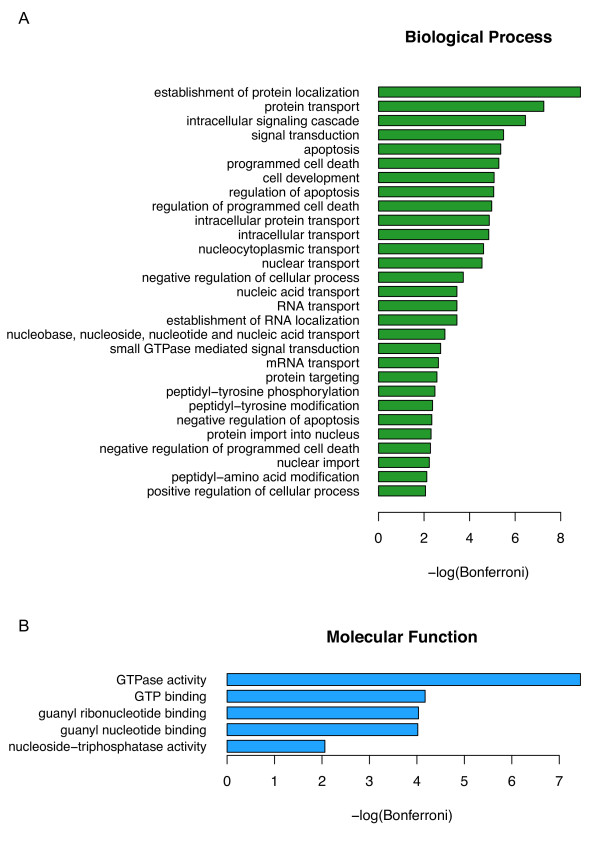
**Significantly enriched Gene Ontology terms in the Human-HIV-1 interaction network**. GO Terms removed at least 5 levels from the root for *(A) *Biological process and *(B) *Molecular function. Bonferroni corrected p-values (α = 0.01) were -*log*_10 _transformed.

Using the CC-filtered predictions, we next examined the function of human proteins predicted to interact with HIV-1 during infection. In this instance, we sought biological process and molecular function GO terms that were enriched among these target proteins. Examining the function of human proteins found in our filtered list of interactions, significant enrichment is observed in the processes of protein transport, nucleic acid transport, signaling, cell death, and post-translational modifications (Figure 5A); all of these processes are known to be manipulated or altered by HIV-1 during infection. During the course of the HIV-1 lifecycle, viral protein and nucleic acids must be transported from one part of the cell to another to ensure viral replication. The Pre-Initiation Complex (PIC), consisting of a number of viral and host proteins and the viral genome, must be transported from the site of viral entry to the nucleus for integration of the provirus. In addition, Env and Vpr are known to play both pro- and anti-apoptotic roles by manipulating host signaling. For instance, there is evidence that HIV-1 may inhibit apoptosis in infected cells to prevent the cell from dying before the virus can replicate and assemble. On the other hand, HIV-1 can also promote apoptosis of immune cells using several pathways; indeed, the progressive destruction of CD4^+ ^T cells is a well known indication of AIDS [27].

Interestingly, all of the significantly enriched molecular function GO terms relate to GTP binding or hydrolysis (Figure 5B). GTPases are involved in a number of host processes that HIV-1 may take advantage of, including nuclear transport and cytoskeletal rearrangements that facilitate viral entry and cellular motility. Statins, a class of drugs that lowers cholesterol levels in the blood, have also been shown to inhibit HIV-1 infection by preventing viral fusion with the cell membrane through a mechanism that involves inhibition of Rho GTPases [28]. In addition, p115-RhoGEF inhibits HIV-1 gene expression through the activation of RhoA [29]. Furthermore, both Rho and Rho kinase play a role in the cellular motility that allows HIV-1 infected monocytes to cross the blood-brain barrier to cause HIV-1 encephalitis [30].

Actin microfilaments of the cytoskeleton are regulated by actin-binding proteins as well as Rho family small GTPases including Rho, Rac, and Cdc42 [31]. IN, RT, and gp41 were all predicted to interact with RhoA, Rac1, and Cdc42 (Figure 4). We found that gp41 has regions of structural similarity with many cytoskeleton related proteins, including erythrocytic spectrin alpha (SPTA1), erythrocytic spectrin beta (SPTB), alpha actinin 4 (ACTN4), alpha actinin 2 (ACTN2), moesin (MSN), Rho-associated coiled-coil containing protein kinase 1 (ROCK1), and arfaptin 2 (ARFIP2). IN resembles NCK adaptor proteins 1 and 2 (NCK1/2), dynactin 1 (DCTN1), and RAS GTPase activating protein 1 (RASA1), among others (Additional File 4). The cytoskeleton has been suggested to be manipulated by HIV-1 during virion fusion, assembly, and budding [31]. HIV-1 movement through the cell can be blocked by drugs that cause depolymerization of microtubules and actin filaments. Actin has also been found within HIV-1 virions, and is incorporated through binding with NC [32]. Thus, our predictions may aid further investigation into the ways in which HIV-1 manipulates the cytoskeleton.

By integrating a variety of high-quality functional data sets in the Literature Filter, we created a smaller interaction map that has the potential to provide a physical interaction context for a number of experimental findings. As an example, retroviral budding is known to involve members of the endosomal sorting complexes (ESCRTs). The ESCRT complexes normally induce the formation of multivesicular bodies in the endosome, but can be recruited to the plasma membrane by Gag to aid in viral budding. Many members of the ESCRT machinery appear in our results, including VPS4A, STAM2, EEA1, RAB5A, and TSG101 [1]. Early endosomal autoantigen 1 (EEA1) is recruited to early endosomes by Rab5 and phosphatidylinositol 3-phosphate [33]. Our results show that gp41 and Gag p2 may interact with RAB5A, since they are structurally similar to EEA1 (Figure 4, Additional Files 1 and 3). EEA1 contains a FYVE domain and colocalizes with human hepatocyte growth factor-regulated tyrosine kinase substrate (Hrs) protein [33,34]. Gp41 is also known to interact with AP1G2, an important component of clathrin-coated vesicles. AP1G2 interacts with RAB5A and provides further support for the possibility that gp41 interacts physically with RAB5A, but through a potentially different structural motif [35]. The Gag p6 protein is a known mimic of Hrs, and like Hrs can recruit TSG101, which is required for the formation multivesicular bodies (MVBs) and viral budding [36]. Gag p2, as well as a model of gp41, show structural similarity to the human protein CEP55, which recruits TSG101 to the thin membrane that separates the daughter cells, where it is needed for the final separation of two cells [37]. Our results suggest that gp41, IN, and the p2 region of Gag may all be able to interact with TSG101 (Figure 4, Additional File 4). Overall, interaction predictions are supported by a variety of studies implicating host mechanisms of vesicle formation in HIV-1 infection.

### Additional method assessment

To further assess our predictions, we determined how many known interactions, curated within either HHPID or PIG, could have possibly been predicted using our method and the available data. First, in order for our approach to suggest a possible HIV-human interaction, the HIV protein must be represented among the crystal structures from PDB that are included in the Dali Database. In addition, any host factors predicted to interact with HIV-1 must have at least 1 known interaction with another human protein, and to be considered further, each of these must also have representative structures within Dali. Finally, in this work we included only those proteins that have been implicated in playing a role in HIV-1 infection through RNAi studies or studies of the protein composition of the virion. Since we removed any human target proteins that did not pass the Literature Filter, we did not make predictions for human proteins not mentioned in previous studies.

A total of 319 known host-pathogen interactions satisfied these criteria. Sixty-two of these interactions (~19%) were predicted by our methodology, and are the set of predictions considered to be true positives (shown in Table 3). We also investigated how many of these possible interactions could have been found after using the cellular component filter, and determined that only 166 known interactions met the additional criterion of being annotated to the same cellular component. Within this set, our method found 31 of these (~19%). This result suggests that while the number of interactions considered was decreased by considering cellular localization, the number of true positive predictions did not improve. Obviously, without experimental validation we cannot determine whether the CC filter led to better prediction accuracy within the set of predictions not previously described in the literature or elsewhere. It is clear, however, that GO cellular component annotation is incomplete and the lack of shared annotation does not completely exclude the possibility that two proteins may interact; inclusion of the CC filter did double the percentage of true positives predicted when considering unknown potential interactions as well as those previously known.

**Table 3:** Method evaluation

Database Evaluation		
	**Before CC filter**	**After CC filter**

Predicted True Positives	62	31

Possible True Interactions	319	166

Percent Found	19.44%	18.67%

Comparison of the number of known interactions predicted to the number of known interactions that could have theoretically been found using the available data.

As an additional form of assessment, we investigated how often we could expect to find previously known interactions by chance alone. Starting from proteins in HPRD, we found that ~0.17% of the known interactions could be found at random (see Methods). Cellular Component filtering of these random predictions gave a slight improvement with an average of 0.29% true positives (Table 4). Using only HPRD human target proteins that pass the Literature Filter increased the true positive accuracy of random predictions to 0.57%. This value can be compared to the value of 2.89% indicated in Table 2. When these random predictions were also run through the CC Filter, an average of 1.03% true positives were found (Table 4) versus a 6.18% when using our method (Table 2). Thus the Literature Filter and the CC Filter improved the accuracy of the true positive predictions individually, and to an even greater extent when combined. However, even with both filters, at best ~1% of the random predictions were found to be true positives, further indicating that incorporating structural information generates predictions with enhanced accuracy and biological validity.

**Table 4:** Accuracy of Random Predictions

Random Predictions		
**Filtering**	**Mean Accuracy**	**Standard Error**

None	0.166%	2.79e-3%

CC	0.286%	6.09e-3%

Lit	0.567%	4.84e-3%

Lit CC	1.030%	1.07e-2%

Shown are the mean percent of true positives and standard error of the mean for random predictions without any filtering (None), CC Filtering alone (CC), Literature Filtering alone (Lit), and both Literature and CC Filtering (Lit CC).

### Overlap with other studies

**Figure 6:**
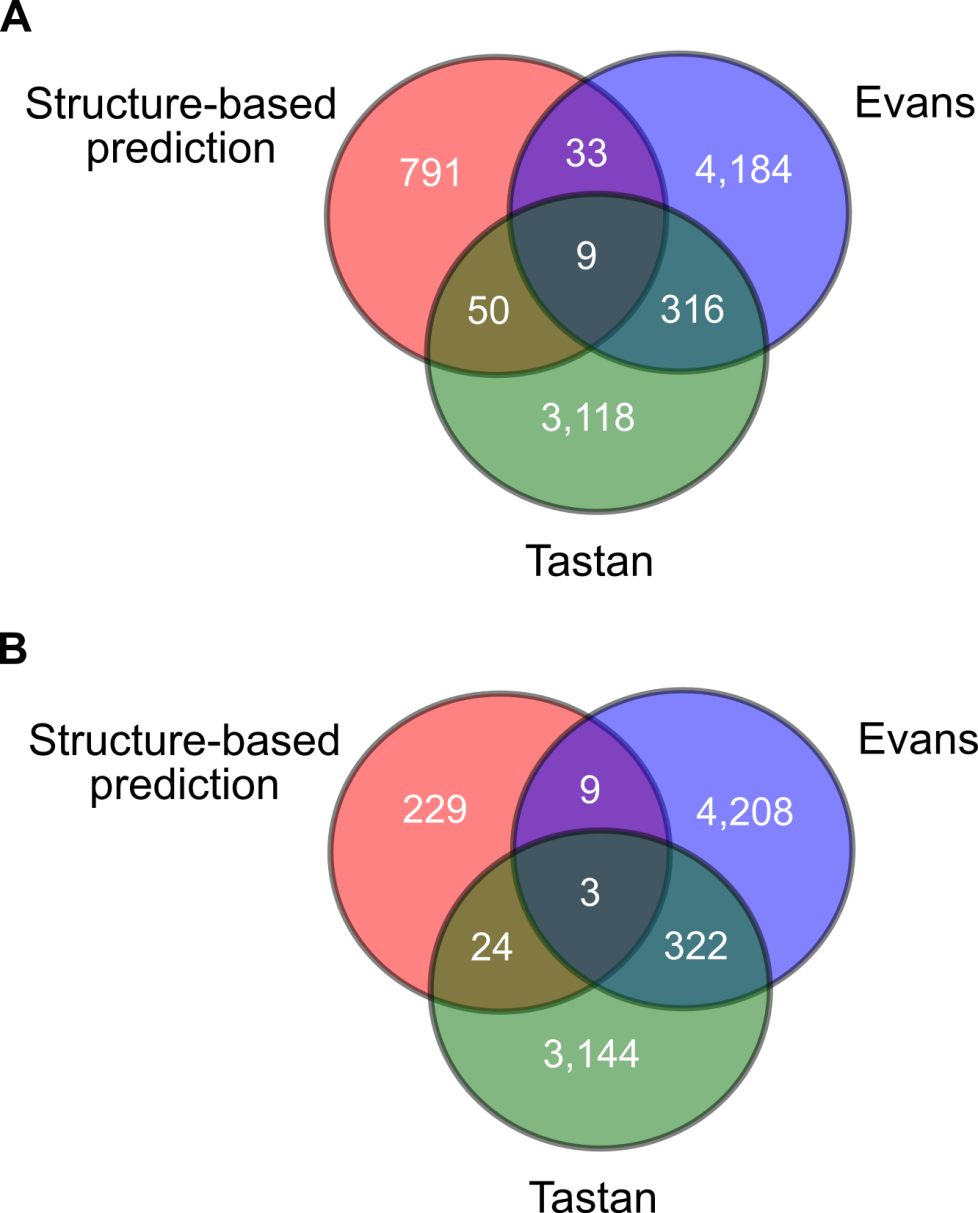
**Overlap with previous studies**. Venn diagrams of the overlap between between our method and previous computational studies by Evans et al. and Tastan et al. (A) with Literature filter and (B) with Literature and CC filter [9,10].

We also compared our predictions to those made by two previous computational studies predicting protein-protien interactions between HIV-1 and humans, namely the studies by Evans et al. and Tastan et al. [9,10]. Since these investigations reported their results in terms of genes, we compared them to our predictions as counted by gene, to find interactions predicted by multiple methods (Figure 6). We did not find a high degree of overlap between the predictions made by the various studies. This was not surprising, as even large-scale experimental protein interaction studies typically show little overlap in their results. Furthermore, the methodology used to generate the predictions differed significantly between studies. Our method used structural similarity to predict interactions, whereas Evans et al. looked for the presence of sequence motifs and counter domains and Tastan et al. integrated a variety of information, including information from GO, properties of the human interactome, and sequence motifs [9,10]. There are a greater total number of shared predictions between Evans et al. and Tastan et al. than between our results and either one of the others. This may be due to the fact that Tastan et al. incoportated Eukaryotic Linear Motifs (ELMs) and binding domains, the key predictor used in the work of Evans et al., as one of the features used in their prediction method. In addition, the other two studies had a larger number of predictions overall. Approximately 7% of the predictions by Tastan et al. were found in the study by Evans et al. Approximately 5% of our predictions (Literature and CC filtered) were found by Evans et al. and 10% were shared with Tastan et al.

There were a few predictions that were shared between all methods. For our results before CC filtering, we found that there were 9 interactions predicted by all three methods (Figure 6A). Of these, four were determined to be true positives in our results: RT and MAPK1, gp41 and LCK, gp41 and PTPRC, and IN and PRKCH. The other five interactions (RT and PIN1, p2 and MAPK1, p2 and YWHAZ, gp41 and PLK1, gp41 and MAPK1, gp41 and CLTC, IN and XPO1, and IN and YWHAZ) are not known to occur, and may be good candidates for further investigation since they were predicted by three diverse methods. After we filtered our predictions by shared cellular components, three predictions were still common between all three studies, gp41 and LCK, gp41 and PLK1, IN and XPO1, one of which is a known interaction (Figure 6B). In summary, although few predictions were shared by all three studies, a large proportion of them are already known to occur, suggesting that the others may be worthy of high priority in future experimental efforts.

## Conclusions

We have generated a map of potential protein-protein interactions between HIV-1 and its human host. The computational methodology used to create this map is based on the assumption that proteins with similar structures will share similar interaction partners. Thus HIV-1 proteins having a structure similar to one or more human proteins may potentially "plug in" to the host protein interactome at these points; providing the interface through which manipulation of downstream host processes can occur. From previous literature, many human proteins are known to play some role in HIV-1 infection. However, in most cases the nature of this role is unknown. Here, we provide specific predictions of how these human proteins may influence viral infection, namely by interacting with certain HIV-1 proteins.

In principle, our approach is applicable to any host-pathogen system with known protein structures. HIV-1 has a small proteome, with most of its protein structures at least partially determined. In addition, HIV-1 also has a large set of identified interactions that can be used for model validation. While few pathogens currently have such rich data sets, continued progress in protein structure determination will help to remedy such deficiencies.

Identification of points of modulation between a host and pathogen requires multiple lines of evidence. Computational methods can help integrate these data, providing a promising avenue for the discovery of novel host-pathogen interactions mediated by structural similarities as well as enhancing our understanding of functional relationships characterized through modern screening methods such as siRNA. Knowledge of the protein interaction network between the pathogen and human will not only further our basic understanding of pathogen survival mechanisms, but may also provide clinical targets to combat infectious disease.

## Methods

### Data Sources

We used the Dali Database for structure comparisons (downloaded in January 2009), and the Human Protein Reference Database (HPRD), HHPID and PIG for protein interactions (downloaded February, July and June 2009, respectively) [16-18,25,38]. The literature sources and various databases used each have their own system of identifiers. PDB codes obtained from Dali were mapped to their corresponding taxonomy and Uniprot accessions using data from the SIFTS initiative [11,39,40]. Other identifier mappings were carried out using DAVID Gene ID Conversion or Uniprot ID mapping [41-43]. Network diagrams were created in Cytoscape [44]. Images of protein structures were created in MacPyMol [45].

### Determination of structural similarity between HIV-1 and host proteins

We used the Dali database to ascertain structural similarity. Dali compares the 3D structural coordinates of two PDB entries by alignment of alpha carbon distance matrices, allowing for differences in domain order, and produces a structural similarity score [11,16,17]. The Dali Database includes structural comparisons where proteins from PDB90, a subset of the PDB where no two proteins share more than 90% sequence similarity, were used as queries against the full PDB [46]. For this study, we took into consideration all human proteins that were listed in the Dali database as being similar to an HIV-1 protein (NCBI Taxonomy ID: 11676) and having a z score above 2.0, with the HIV-1 protein being either the query or the hit. We refer to these human proteins as "HIV-similar" proteins. No proteins of unknown structure were considered.

### Interaction Prediction

We found known interactions between HIV-similar proteins and target human proteins, using data from the HPRD database, which contains literature curated interactions between pairs of human proteins [18]. For each HIV-similar protein, we predict that the target proteins, which are known to both interact with the HIV-similar protein and pass the Literature Filter, might also interact with the corresponding HIV-1 protein. Therefore, interactions between the HIV-similar and the human target proteins were mapped directly to the corresponding HIV protein.

### Filtering

To reduce the number of predictions as well as add information from functional studies, predictions were filtered based on previous implication of the human protein's involvement in the HIV-1 infection process. One criterion was presence of the host protein in the HIV-1 virion. Host proteins known to be incorporated into or onto HIV-1 during budding were taken from several literature sources [23,24,47]. The presence of host proteins in or on HIV-1 may be a result of specific recruitment and serve a functional role, may result from localization of the protein near the site of budding, or may simply occur by chance. Predicted interactions between HIV-1 proteins and human proteins that are incorporated into the HIV-1 virion were retained. In addition, any human protein that is incorporated into the virion and is itself structurally similar to an HIV-1 protein was also included as a possible interaction.

Another filtering criterion was the host protein's essentiality for HIV-1 infection. Recently, several large-scale experiments using siRNA or shRNA knockdowns to identify host proteins involved in the HIV-1 life cycle have been published [19-22]. The probe ids of the genes implicated by Yeung et al. were mapped to their Entrez Gene IDs using the appropriate Affymetrix annotation file http://www.affymetrix.com/products_services/arrays/specific/hgu133plus.affx#1_4[22]. This filter is referred to as the "Literature Filter." Host proteins that were implicated in at least one of these studies as having a possible role in HIV-1 infection or replication, and which are also known to interact with an HIV-similar protein, were predicted to interact with an HIV-1 protein in the final predicted network. 

To create a smaller and potentially more reliable list for further experimental validation, we further filtered the predictions based on shared sub-cellular localization. The Cellular Component (CC) Filtered dataset contains interaction predictions where the two proteins share Gene Ontology (GO) cellular component annotation. Pairs of HIV-1 and human proteins predicted to interact were only included in this dataset if both proteins were annotated by DAVID as being present in the same cellular compartment [41,42]. Pairs with only the terms "cell" and "cell part" in common were excluded due to a large number of such pairs and the relative lack of specificity of these high level terms.

### Validation of Predictions

Since within Dali there may be multiple PDB structures representing the same protein, there is some redundancy in the interaction predictions. In certain cases, multiple PDB structures for the same HIV-1 protein were found to be similar to multiple PDB structures for an HIV-similar protein, leading to the same interaction predictions. Therefore, the predictions were counted as unique pairs of Uniprot accessions. In addition, for ease of viewing the predicted interactome, each node representing an HIV-1 protein is labeled with the protein name while each human protein is represented by Entrez Gene ID. To determine the correct mapping of PBD codes to HIV-1 proteins, the molecule name associated with each PDB chain was searched for keywords indicating the protein, with ambiguous cases treated on an individual basis. For example, PDB molecule names containing the word "capsid" but not "nucleocapsid" were assigned to the node "capsid." Furthermore, molecule names indicating polyproteins, such as those containing the phrase "gag-pol" were checked individually to determine which specific part of the polyprotein was represented by the entry. Two PDB structures were found to represent more than one mature HIV-1 protein: 1l6nA contains both capsid and matrix, while 1bajA contains capsid and p2 [48,49]; these structures are represented as "capsid, matrix" and "capsid, p2" respectively. When counting predictions at the gene level, we considered pairs of HIV-1 node names and human target Entrez Gene IDs.

To determine which predictions are true positives, PIG and HHPID entries for the predicted human interactors were examined to see if they contained the HIV-1 protein they were predicted to interact with [25,38]. These interaction databases consist of PPIs curated from the literature. HHPID uses keywords to characterize the different types of interactions listed in this database. Since this work attempts to predict physical interactions, only entries with keywords representing direct interactions were included [9]. The Uniprot accessions associated with the HIV-1 protein PDB entry, in the case of PIG, or the Entrez Gene ID mapped to that Uniprot accesion, in the case of HHPID, was checked to see if it was present as an already known interaction of the human protein.

### GO Term Enrichment

The Gene Ontology (GO) provides a system of terms to consistently describe and annotate gene products [50]. GO term enrichment was performed using the DAVID Functional Annotation Chart tool. The GO is organized as a tree structure, with terms becoming more specific as distance from the root increases. Therefore, to avoid very general and uninformative GO terms, only those that are found at least 5 steps removed from the overall root of GO were considered. The p-values were corrected for multiple testing using the Bonferroni procedure and transformed by taking the -*log*_10 _for easier visualization.

### Computational evaluation

Two forms of computational validation of the method were conducted. As it is not possible to predict all known interactions due to lack of protein structures, as well as other factors, we first determined the largest set of known interactions that it is theoretically possible to predict using our approach. To do this, we first determined the sets of all proteins that could be considered. This includes the set of all HIV-1 proteins in Dali (HIV set), the set of all human proteins that are represented in both Dali and HPRD (possible HIV-similar set), and the set of all human proteins in HPRD that are known to interact with at least one protein in the possible HIV-similar set as well as pass the literature filter (possible target set). Next, every pairwise combination of proteins in the HIV set and the possible target set was checked to see if it represented a known interaction curated in HHPID or PIG. The resulting number of true interactions that could have been found by the method was compared to the number of true positives that were actually found, both before and after filtering by cellular components.

In the second approach, actual prediction results were compared to predictions based on randomly selected HIV-human protein pairs. The HIV-1 proteins were chosen from the 69 Uniprot accessions represented at least once by structures in the Dali Database. For human proteins, two different sets of human Uniprot proteins were created, one containing all the proteins in HPRD, and the other containing the subset of human proteins that also passed the Literature Filter. The set of all human proteins in HPRD consisted of 8582 proteins and was used to see the accuracy of purely random predictions, while the second set of 830 proteins was used to observe the effect of the Literature Filter.

Since the structural similarity step was omitted, the predictions based on a human protein being similar to an HIV-1 protein and incorporated into the virion could not be simulated with the random selection procedure. We found that if we excluded this class of predictions from our real results, the number of unique predictions made was reduced to 2139, but all 62 true positives were still included. Therefore, we randomly selected 2139 pairs of HIV-1 proteins and human proteins from the entire HPRD, and a second set of 2139 pairs of HIV-1 proteins and Literature Filtered human proteins for evaluation. Next, any known interactions between the randomly chosen pairs were found using HHPID and PIG. Additionally, both the unfiltered and Literature Filtered random predictions were then subjected to the CC Filter to gauge the improvement due to this step of the method. The CC Filter reduced the number of predictions to a variable degree, depending on how many of the random predictions were annotated with the same GO cellular component term. The entire procedure was repeated 1000 times. The mean and standard error of the mean for each of the four variously filtered random prediction sets was calculated using R. The distributions of random predictions after Literature Filtering were approximately normal, so one-sided single sample t-tests were performed to determine if the method performed significantly better than random. In addition, we performed Wilcoxon signed-rank tests that do not make assumptions about normality. When comparing our results to random predictions that had undergone the same filtering steps, either the Literature Filter or both the Literature and CC Filters, the p-values were less than 2.2e-16 for all statistical tests. In addition, even when performing the randomization procedure 10000 times, none of the randomly selected interaction sets had a true positive rate higher than that observed in our results, suggesting a p-value of no greater than 0.0001.

To compare our predictions to those made by Evans et al. and Tastan et al., we found the intersection of the prediction sets, counted by HIV-1 protein name and human Entrez Gene ID [9,10]. Since each study used different names for the HIV-1 proteins, we had to map the naming schemes to each other to find common predictions. For example, Evans et al.'s "CA" and "GAG" and Tastan et al.'s "gag_capsid" and "gag_pr55" were mapped to our "capsid." Proteins for which we made no predictions, such as Rev, were not mapped to anything in our results, but were converted between Evans et al. and Tastan et al. to find overlap between these two studies.

## Competing interests

The authors declare that they have no competing interests.

## Authors' contributions

JMD and SMG conceived and designed the study, JMD performed the research. JMD and SMG analyzed the results and wrote the manuscript. Both authors read and approved the final manuscript.

## Supplementary Material

Additional file 1 **Dali Similarities**. Structural Similarities between HIV-1 and human proteins from the Dali Database. Column descriptions: HIV PDB code- PDB code of an HIV-1 protein in Dali, HIV Molecule Name- description of the HIV protein taken from the PDB molecule name, HIV GeneID- the Entrez GeneID of the HIV protein if available, HIV Uniprot- Uniprot accession of the HIV protein, human PDB code- PDB code of a structurally similar human protein, human Molecule Name- description of the human protein taken from the PDB molecule name, human GeneSymbol- the Entrez Gene Symbol of the human protein, human GeneID- the Entrez GeneID of the human protein if available, human Uniprot- Uniprot accession of the human protein, Structural Similarity Score- the z-score for the similarity between the HIV and human protein. This file is provided in tab-separated text format and may be viewed in a text editor or Excel. Click here for file

Additional file 2 **Interaction Predictions**. Interaction predictions, before the CC Filter. Column descriptions: HIV PDB code- PDB code of an HIV-1 protein in Dali, HIV node name- protein name used to represent the HIV protein in the interaction network, HIV-sim Human PDB code- PDB code of a human protein similar to the HIV protein, HIV-sim Human Gene Symbol- the Entrez Gene Symbol of the human protein that is similar to the HIV protein, HIV-sim Human GeneID- the Entrez GeneID of the human protein that is similar to the HIV protein, HIV-sim Human Uniprot- the Uniprot accession of the human protein that is similar to the HIV protein, Human node Gene Symbol- the Entrez Gene Symbol of a human protein predicted to interact with the HIV protein. The Gene Symbol was used to represent the human protein in the interaction network, Human interactor GeneID- the Entrez GeneID of a human protein predicted to interact with the HIV protein, Human interactor Uniprot - the Uniprot accession of a human protein predicted to interact with the HIV protein, Source Datasets- paper(s) with support for a role of the human protein in HIV infection, True Positive?- whether or not the predicted interaction is already represented in PIG or NIAID, Type-whether the prediction was made because the human protein is known to interact with an HIV-similar protein or because the human protein is HIV-similar itself and known to be incorporated into the HIV virion, NIAID- HIV proteins listed in NIAID as interacting with the human protein, PIG- HIV proteins listed in PIG as interacting with the human protein. This file is provided in tab-separated text format and may be viewed in a text editor or Excel. Click here for file

Additional file 3 **Full Prediction Network**. HIV-1 proteins that resemble human proteins are predicted to interact with the known interactors of the mimicked protein. The human proteins included in the prediction set have a supported role in HIV-1 infection or replication, either because they are incorporated into the HIV-1 virion or their reduced expression is known to prevent HIV-1 infection (node line color corresponds to source). Red lines represent predicted interactions that are already known to occur. This is an image file in .png format. Click here for file

Additional file 4 **Interaction Predictions after CC Filter**. Interaction predictions after the CC Filter. Column headers are as in Additional file 2, with the addition of the last column, CC in common- list of GO cellular component terms annotated to both the HIV and human proteins. This file is provided in tab-separated text format and may be viewed in a text editor or Excel. Click here for file

Additional file 5 **HIV-1 Protein Identifiers**. The PDB codes and Uniprot accessions that correspond to each HIV-1 protein are given. This file is provided in tab-separated text format and may be viewed in a text editor or Excel. Click here for file
