# Supplementary figures and images for: Structural similarity-based predictions of protein interactions between HIV-1 and Homo sapiens
Source: Virol J. 2010 Apr 28;7:82. doi: 10.1186/1743-422X-7-82 (PMC2877021; doi:10.1186/1743-422X-7-82)

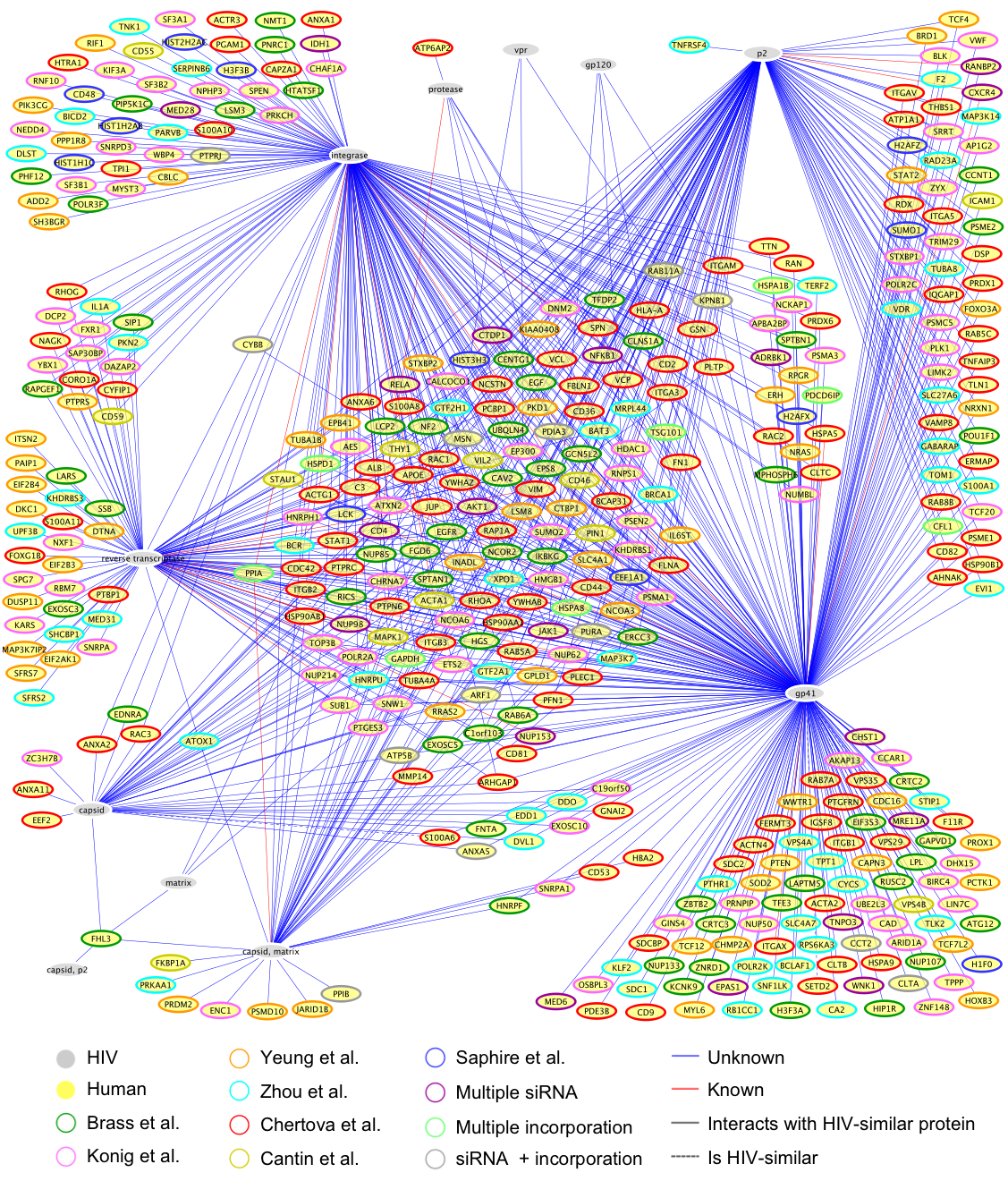

Supplement: Additional file 3 — Full Prediction Network. HIV-1 proteins that resemble human proteins are predicted to interact with the known interactors of the mimicked protein. The human proteins included in the prediction set have a supported role in HIV-1 infection or replication, either because they are incorporated into the HIV-1 virion or their reduced expression is known to prevent HIV-1 infection (node line color corresponds to source). Red lines represent predicted interactions that are already known to occur. This is an image file in .png format. [file 1743-422X-7-82-S3.PNG]
